# Plasma membrane localization of the GFL receptor components: a nexus for receptor crosstalk

**DOI:** 10.1007/s00441-020-03235-4

**Published:** 2020-08-07

**Authors:** Christopher R. Donnelly, Brian A. Pierchala

**Affiliations:** 1 Department of Anesthesiology Center for Translational Pain Medicine, Duke University Medical Center, Durham, NC 27710 USA; 2 Department of Anatomy, Cell Biology & Physiology, Stark Neurosciences Research Institute, Indiana University School of Medicine, Indianapolis, IN 46202 USA

**Keywords:** Ret, GFRα, Plasma membrane, Intracellular trafficking, GDNF, GFLs, TGF-β regulation

## Abstract

The glial cell line-derived neurotrophic factor (GDNF) family ligands (GFLs) comprise a group of four homologous and potent growth factors that includes GDNF, neurturin (NRTN), artemin (ARTN), and persephin (PSPN). The survival, growth, and mitotic activities of the GFLs are conveyed by a single receptor tyrosine kinase, Ret. The GFLs do not bind directly to Ret in order to activate it, and instead bind with high affinity to glycerophosphatidylinositol (GPI)-anchored coreceptors called the GDNF family receptor-αs (GFRαs). Several mechanisms have recently been identified that influence the trafficking of Ret and GFRαs in and out of the plasma membrane, thereby affecting their availability for ligand binding, as well as their levels by targeting to degradative pathways. This review describes these mechanisms and their powerful effects on GFL signaling and function. We also describe the recent discovery that p75 and Ret form a signaling complex, also regulated by plasma membrane shuttling, that either enhances GFL survival signals or p75 pro-apoptotic signals, dependent on the cellular context.

## Introduction

In the past, illustrations detailing neurotrophic factor signaling pathways often indicated relatively simplistic interactions between well-established ligand-receptor pairs, with each complex separately leading to the activation of downstream signaling cascades controlling a variety of cell fates. Within the neurotrophin family, for example, nerve growth factor (NGF) binds to TrkA, brain-derived neurotrophic factor (BDNF) and neurotrophin-4 (NT-4) bind to TrkB, and neurotrophin-3 binds to TrkC, and all four ligands can bind with reduced affinity to p75 (Reichardt 2006). However, as our understanding of neurotrophic factor signaling has evolved over the last several decades, we have come to appreciate that a highly complex set of interactions exists between neurotrophic factor receptors, as well as other classes of receptors, and the cellular context of these interactions ultimately determines the fate of neurons during neural development (e.g., survival, death, differentiation, axon growth). Interactions between neurotrophic factor receptor signaling pathways have been demonstrated in a variety of cellular contexts, including (1) indirectly, via integration of shared downstream signaling pathways; (2) directly, through ligand-dependent or ligand-independent interactions at the cell surface; and (3) through mechanisms involving receptor sorting, trafficking, or membrane localization. Here we provide a brief overview of neurotrophic factor receptor crosstalk with an emphasis on Ret, the receptor tyrosine kinase for the glial cell line-derived neurotrophic factor (GDNF) family of ligands (GFLs), for which all three of these mechanisms has recently come to light.

The GFLs are a family of neurotrophic factors distantly related to the transforming growth factor-β (TGF-β) superfamily, consisting of four homologous members: GDNF, neurturin (NRTN), artemin (ARTN), and persephin (PSPN). The GFLs exert their functions by first binding as homodimers to lipid raft–localized glycophosphatidylinositol (GPI)-linked co-receptors known as the GDNF family receptor-αs (GFRαs), which then recruit and engage Ret. Each GFL has a preferred GFRα co-receptor, with GDNF preferentially binding to GFRα1, NRTN to GFRα2, ARTN to GFRα3, and PSPN to GFRα4, although cross-binding can occur (Airaksinen and Saarma 2002). Of note, GFL signaling is unique: Ret remains the only RTK identified to date which does not bind its ligands directly but instead requires a co-receptor for activation. This peculiarity thus confers additional opportunities to fine-tune GFL signaling through dynamic regulation of both Ret and GFRαs. Since the initial discovery of GDNF-Ret signaling as a critical regulator of kidney and enteric nervous system development in 1996 (Pichel et al. 1996; Sanchez et al. 1996; Schuchardt et al. 1994), the biological functions of Ret have been greatly expanded. In the nervous system, GFL-Ret signaling is now appreciated to play a critical role in the development of several populations of dorsal root ganglion (DRG) somatosensory neurons (Luo et al. 2007, 2009; Molliver et al. 1997) as well as geniculate oral sensory neurons (Donnelly et al. 2017), spinal motor neurons (Dudanova et al. 2010; Gould et al. 2008; Haase et al. 2002), postganglionic sympathetic and parasympathetic neurons (Durbec et al. 1996; Enomoto et al. 2001; Enomoto et al. 2000), orofacial trigeminal nociceptors (Donnelly et al. 2019), and even other populations as well. Ret has recently been found to be activated by growth differentiation factor 15 (GDF15), a metabolic regulator that binds to the co-receptor GFRAL and together engages Ret (Mullican et al. 2017; Yang et al. 2017). Whether Ret is the predominant signal-transducing receptor component underlying the effects of GDF15 on food intake and obesity is under investigation. Beyond normal physiological function, aberrant Ret signaling is also implicated in numerous cancers (Plaza-Menacho et al. 2014). Thus, an appreciation of the regulatory mechanisms governing GFL-Ret signal transduction has the capacity to inform our understanding of a variety of different developmental and disease processes.

### TGF-β regulation of GFL signaling through GFRα1 surface localization

**Fig. 1:**
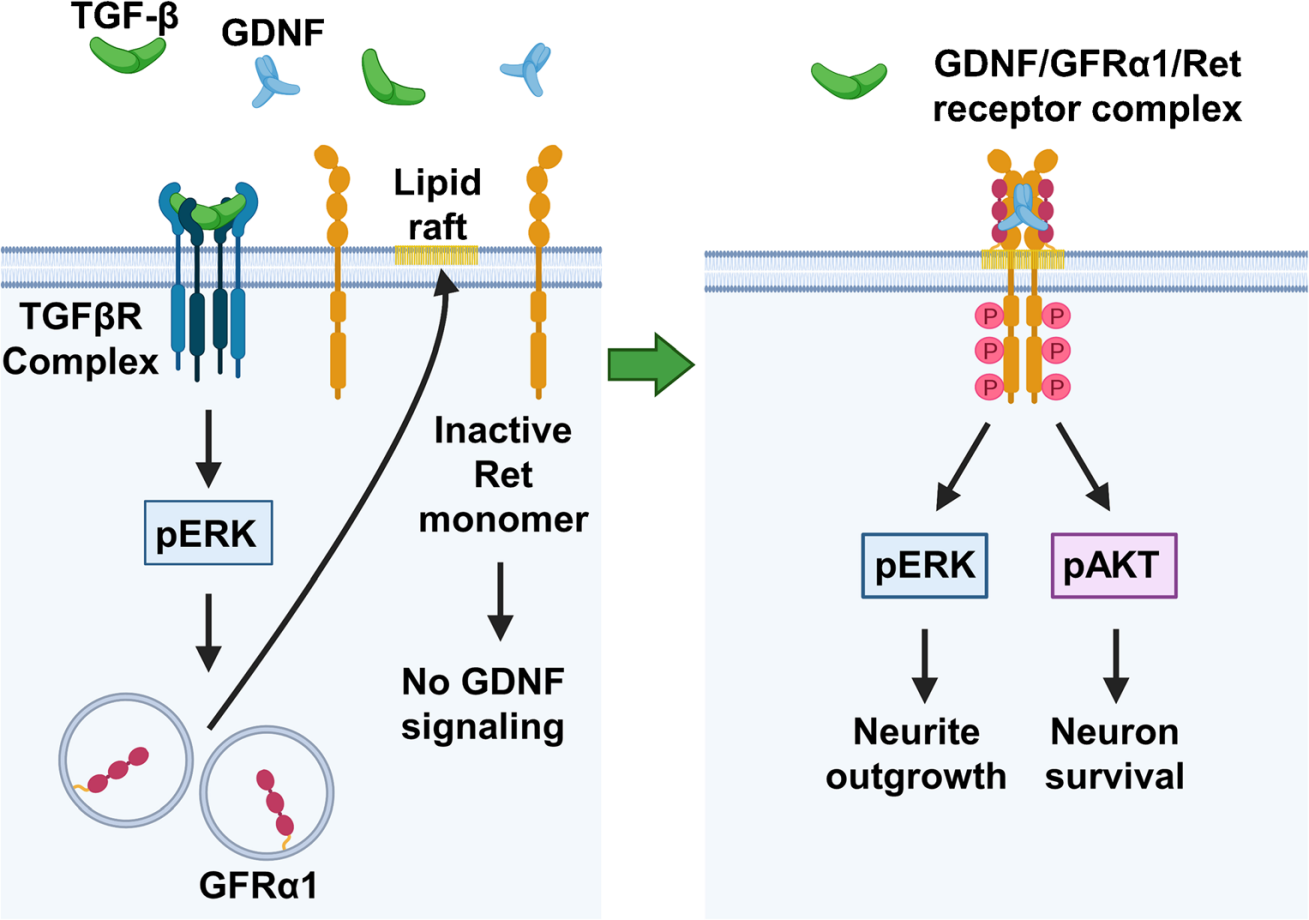
TGF-β regulation of GDNF-Ret signal transduction through GFRα1 cell surface localization

Given the important biological functions carried out by the GFLs, it is perhaps not surprising that several mechanisms exist to precisely regulate GFL signaling through cell surface routing and membrane localization of the GFL receptor components. Interestingly, TGF-β has an important role in the trophic functions of GDNF in developing neurons, both in vitro and in vivo. Krieglstein et al. demonstrated that GDNF-mediated survival of several developing peripheral and central neuron populations in vitro required the addition of TGF-β, and moreover, TGF-β neutralizing antibodies could abolish GDNF-mediated neuronal survival (Krieglstein et al. 1998). In an in vivo model of adrenomedullectomy, removal of the adrenal gland induces the degeneration of preganglionic sympathetic neurons in the intermediolateral column of the spinal cord, and this degeneration can be reduced by the application of GDNF. Interestingly, the neuroprotective effect of GDNF is abolished by the co-treatment of TGF-β neutralizing antibodies with GDNF (Schober et al. 1999). Consistent with these models, co-application of GDNF and TGF-β promotes the survival of developing sympathetic neurons of the chicken ciliary ganglion (CG), while neither factor on their own is able to promote survival (Peterziel et al. 2002). Of note, these effects are blocked by extracellular signal-regulated kinase (ERK) inhibition, but not by inhibition of the PI3 kinase (PI3K) pathway (Peterziel et al. 2002). While CG neurons are unresponsive to physiological concentrations of GDNF at baseline, TGF-β pretreatment for 3 h is sufficient to induce GDNF responsiveness without requiring TGF-β to continue to be present along with GDNF. In fact, after pretreatment with TGF-β, the complete elimination of TGF-β using function-blocking antibodies in combination with GDNF does not alter the survival effects of GDNF (Peterziel et al. 2002). Mechanistically, TGF-β induces the cell surface localization of GFRα1 without affecting either GFRα1 mRNA or protein levels (Peterziel et al. 2002). NRTN, in contrast, was found to promote the survival of developing CG neurons in the absence of TGF-β, and disrupting TGF-β signaling using a pharmacological inhibitor of TGF-β Receptor 2 (Tgfrb2) failed to attenuate NRTN-mediated survival (Peterziel et al. 2007). Consistent with these data, TGF-β induced the recruitment of GFRα1 to the cell surface but did not recruit GFRα2, which was already abundantly localized to the cell surface (Peterziel et al. 2007). Taken together, TGF-β is uniquely involved in GDNF signal transduction via a TGF-β/Tgfrb2/MAPK pathway that promotes the trafficking of GFRα1 to the plasma membrane (Fig. 1). To date, it remains unknown whether TGF-β affects localization of the other two GFL receptor complexes (e.g., Ret, GFRα3, GFRα4) and mechanistically how TGF-β-mediated MAPK activation triggers the movement of internal stores of GFRα1 to the cell surface.

### Shutting off the signal: GDNF, GFRα1, and Ret trafficking by SorLA

Although considerable progress has been made in our understanding of GFL receptor signaling and its downstream consequences, less is known about how ongoing GDNF-GFRα1-Ret signaling is extinguished at the molecular level. On this note, the sorting receptor SorLA was recently demonstrated to have a critical role. Using heterologous cell lines, it has been reported that the proteolytic processing of proGDNF to mature GDNF that occurs during transit through the secretory pathway requires SorLA (Geng et al. 2011). SorLA binds directly to both proGDNF via the prodomain and to mature GDNF, although the association with mature GDNF is lost at the acidic conditions present in the Golgi apparatus, whereas SorLA binding to proGDNF was retained (Geng et al. 2011). In addition, SorLA regulated the secretion of GDNF from the regulated secretory pathway stimulated by synaptic activity, but not GDNF release from the constitutive pathway (Geng et al. 2011). Following these observations, it was discovered that SorLA binds to GFRα1 with very high affinity and that SorLA is capable of binding to GFRα1 and GDNF simultaneously (Glerup et al. 2013). Interestingly, SorLA does not bind to the other GFLs, indicating that this mechanism selectively regulates GDNF signaling (Glerup et al. 2013). Upon binding, SorLA mediates internalization of GDNF and GFRα1 and promotes GDNF routing to lysosomes for subsequent degradation, while the GFRα1/SorLA complex escapes degradation. In addition, the SorLA/GFRα1 sorting complex promotes the endocytosis of Ret, even in the absence of GDNF, thereby sequestering it from subsequent activation and signal transduction (Glerup et al. 2013). Consistent with these findings, cultured hippocampal neurons lacking SorLA exhibited elevated cell surface levels of GFRα1, and GDNF was retained on the cell surface, in contrast to the rapid internalization of GDNF and its degradation in neurons expressing SorLA (Glerup et al. 2013). Importantly, *SorLA*^*−/−*^ mice display anatomic and functional abnormalities of dopaminergic midbrain circuits, such as elevated levels of GDNF in the midbrain and striatum, a marked loss of nigrostriatal connectivity, a blunted response to amphetamines, significant hyperactivity, and reduced anxiety levels (Glerup et al. 2013). These studies raise the possibility that SorLA could function to restrict GDNF/GFRα1/Ret signaling to specific subcellular regions. SorLA is reported to be predominantly located in cell bodies, dendrites, and initial segments but is excluded from distal axons and terminals (Glerup et al. 2013; Posse De Chaves et al. 2000). This would result in the clearance of GFRα1 and Ret from the cell surface of the somatodendritic compartment, thereby only allowing for GDNF-mediated Ret activation to occur in axons. Furthermore, the function of SorLA in the regulated secretion of GDNF would allow for the activity-dependent release of GDNF from dendrites while avoiding autocrine activation of Ret in these same regions. Additionally, it is tempting to speculate that the ability of TGF-β to promote the routing of intracellular GFRα1 to the cell surface could be due to an inhibition of SorLA activity.

### Bypassing the surface regulation of co-receptors: Ret activation by GFRαs in *trans*

Cells that express Ret usually also express one or more GFRα co-receptor. Interestingly, GFRαs, especially GFRα1, have considerably more widespread expression compared to Ret-expressing cells. This led to the speculation that GFRαs expressed in Ret-negative cells signal via receptors other than Ret, or that GFRαs, when bound to the appropriate GFL, can activate Ret in *trans* (in *trans* relative to Ret expression), in a cell non-autonomous manner. Evidence for the former possibility has emerged, and NCAM and syndecan-3 are both alternative signal-transducing receptors for GDNF (Bespalov et al. 2011; Paratcha et al. 2003).

Evidence supporting the physiologic role for GFRα function in *trans* has been more complex. The first issue to consider is whether GFRα co-receptors are capable of activating Ret in *trans*. Indeed, several studies indicate that soluble GFRαs, as well as GFRα co-receptors immobilized onto surfaces, such as charged beads, potently activate Ret and support survival, neurite outgrowth, axon guidance, and migration (Ledda et al. 2002; Paratcha et al. 2001). This is especially true in 3-dimensional culture systems such as DRG explants and ex vivo gut systems (Fleming et al. 2015; Patel et al. 2012). Cell lines, primary neurons, and various neural explants release soluble GFRαs into the culture medium, presumably by cleavage of the GPI anchor or by proteolysis, also supporting the possibility that GFRαs are shed from cell surfaces and act in *trans* (Fleming et al. 2015; Paratcha et al. 2001; Tsui et al. 2015). It is difficult to unequivocally prove, however, that the appearance of soluble GFRαs in tissue culture medium is not due to the stress of dissecting/dissociating the neurons or due to cell death ongoing in the cultures resulting in cell lysis and the accumulation of GFRαs in the medium.

In regard to whether *trans* signaling occurs in vivo, this has been most thoroughly investigated for GFRα1, and a transgenic mouse was produced to directly evaluate this possibility. In this mouse, GFRα1 is only expressed in *cis* in neurons that express Ret by transgenically driving GFRα1 in Ret+ cells in a GFRα1 null background, known in the field as the “cis-only” mouse (Ret^GFRα1/+^; GFRα1^−/−^)(Enomoto et al. 2004). Quite remarkably, all of the most dramatic GDNF/GFRα1 functions, such as kidney morphogenesis, enteric nervous system development, and motor axon growth and guidance, were normal despite the extensive *trans* expression of GFRα1 in these regions (Enomoto et al. 2004). Subsequent studies, however, have provided compelling evidence that *trans* signaling by GFRα1 can occur in vivo. Perineural invasion of some cancers is induced by GDNF, and in vivo models of perineural invasion of sciatic nerve are eliminated when Ret is silenced in the transplanted cancer cells (He et al. 2014). Importantly, elimination of GFRα1 in these same cells reduces, but does not eliminate, their invasive properties, suggesting that GFRα1 expressed in the nerve in *trans* is sufficient (He et al. 2014). In another study, when GFRα1 was transgenically overexpressed in the striatum, there was a decrease in the programmed cell death of mesencephalic dopamine neurons that innervate this target, suggesting an enhancement of *trans* signaling (Kholodilov et al. 2011). In the DRG, some rapidly adapting (RA) mechanoreceptors express Ret and GFRα2, and deletion of Ret results in a dramatic loss of the central projections of sensory axons into the dorsal spinal cord (Fleming et al. 2015). Curiously, deletion of GFRα2 results initially in a complete loss of this innervation during early embryonic ages, but this deficit eventually recovers by birth. This is due to a compensation of GFRα1 expressed in the spinal cord that functions in *trans*, along with GDNF, to guide RA axons to their correct location (Fleming et al. 2015). Deletion of GFRα1 alone has no effect on this process, however, suggesting that the ability of GFRα1 to function in *trans* in this context is compensatory and may not normally provide this function.

While GFRα1 cis-only mice do not have identified developmental deficits in the periphery, it was more recently discovered that these mice have a significant loss of GABAergic interneurons in the cerebral cortex (Canty et al. 2009). GABAergic interneurons populate the cortex by migrating tangentially from the ganglionic eminences, a process that requires GDNF and GFRα1 (Pozas and Ibanez 2005). Analysis of cortical interneurons in cis-only mice revealed that there are large regions of the cortex where parvalbumin-expressing (PV+) interneurons were absent, called “PV holes.” Indeed, it appears that these regions are devoid selectively of PV+ interneurons, whereas other classes of GABAergic neurons, such as somatostatin-expressing and calreticulin-expressing neurons, are normal (Canty et al. 2009). Interestingly, this tangential migration does not require Ret or NCAM, but does require the transmembrane heparin sulfate proteoglycan syndecan-3, suggesting that this is more likely to be a deficit in GDNF/GFRα1 function in PV+ neurons rather than a specific loss of *trans* signaling (Bespalov et al. 2011; Pozas and Ibanez 2005). Considering the data collectively, multiple lines of evidence indicate that GFRα co-receptors provided non-cell autonomously that can activate Ret, and *trans* signaling is likely to be involved in developmental functions such as axon growth and guidance, as well as cell migration.

### Neurotrophic factor receptor crosstalk: reciprocal interactions between Ret and TrkA

**Fig. 2:**
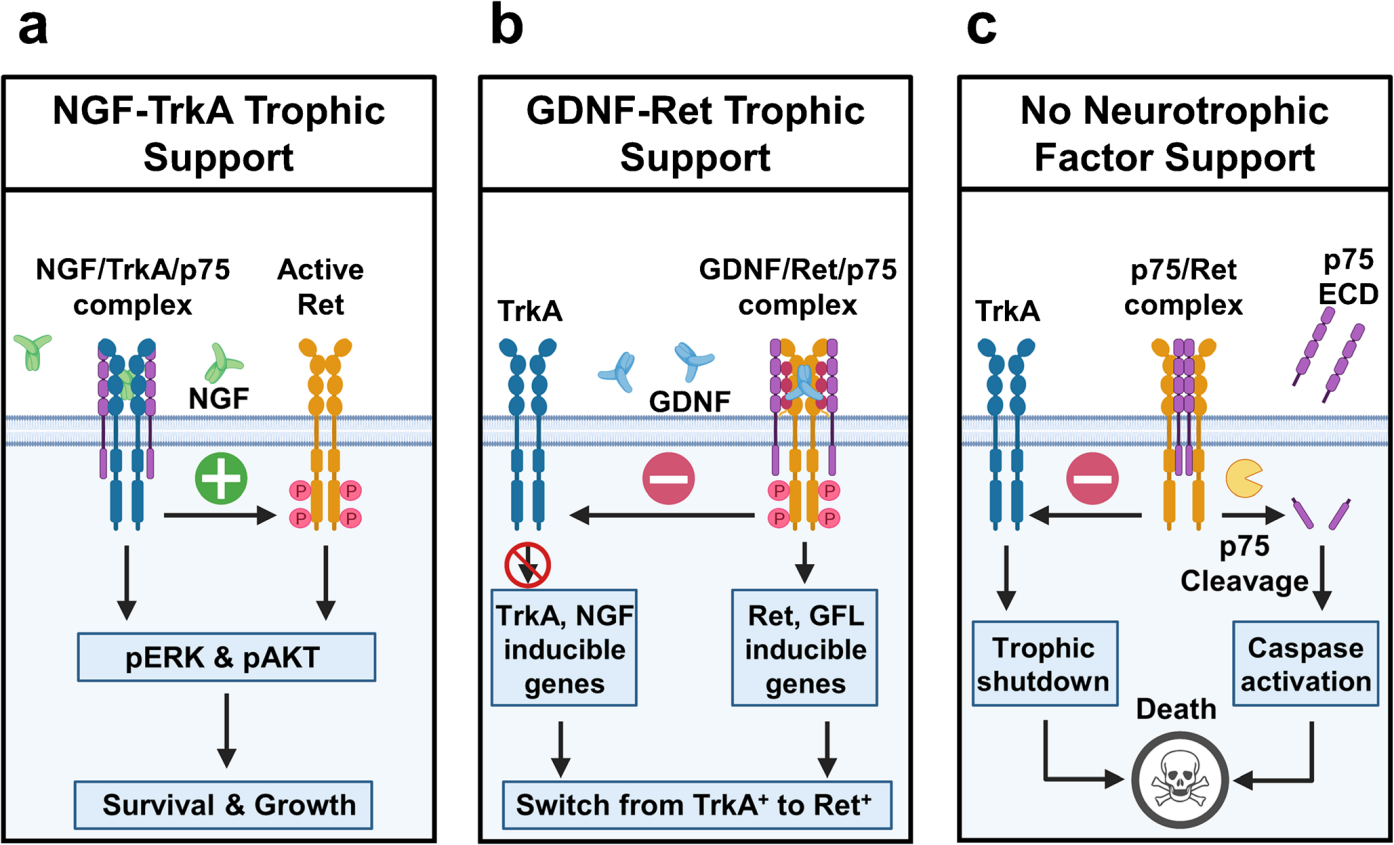
**a** NGF/TrkA/p75 receptor complexes in mature sympathetic neurons promote Ret signaling by retaining autophosphorylated Ret on the cell surface. **b** In developing nociceptors, GDNF/Ret signaling inhibits TrkA signaling triggering the switch from TrkA+ peptidergic nociceptors to Ret+ nonpeptidergic nociceptors. In addition, p75 in these neurons enhances Ret signaling and function. **c** In developing sympathetic neurons, Ret enhances the pro-apoptotic activity of p75 by enhancing TrkA degradation, thereby inhibiting NGF/TrkA survival signaling, while simultaneously enhancing pro-apoptotic death signaling of p75 by promoting its intramembranous cleavage

While initially TrkA and Ret were understood to largely promote the survival of distinct, non-overlapping populations of neurons, it has come to be recognized that considerable crosstalk occurs between these two receptors. During peripheral nervous system development, TrkA and Ret are co-expressed in several populations of peripheral neurons at birth, including sympathetic neurons of the superior cervical ganglion (SCG) and in an actively differentiating population of dorsal root ganglion (DRG) nociceptors (Lallemend and Ernfors 2012). Perinatally, both populations are highly dependent upon target-derived NGF for their survival. Thus, what function does Ret serve in these neurons? Interestingly, phosphorylation of Ret increases with age in sympathetic neurons, both in vitro and in vivo, and in a manner which is both GFL- and GFRα-independent (Tsui-Pierchala et al. 2002). Instead, this effect was found to be dependent on NGF/TrkA signaling and required several hours to induce maximal Ret activation. This NGF-induced Ret phosphorylation only occurred in mature neurons (21 days in vitro; DIV) and not immature sympathetic neurons (5 DIV) that are analogous to neurons in their period of programmed cell death (Tsui-Pierchala et al. 2002). Interestingly, in mature primary sympathetic neurons, Ret contributed to the trophic functions of NGF, as *Ret*^*−/−*^ neurons exhibited somal atrophy, reduced metabolic status, and substantially reduced expression of several critical NGF-inducible genes (Tsui-Pierchala et al. 2002). In a later study, we reported that NGF promotes GFL-independent Ret activation via a mechanism by which NGF suppressed Ret internalization and degradation, leading to an accumulation of autophosphorylated Ret on the cell surface (Pierchala et al. 2007). Mechanistically, NGF promoted monoubiquitination of Ret, which remained on the cell surface, while GDNF activation of Ret promoted its polyubiquitination, which triggered the rapid internalization and degradation of P-Ret (Pierchala et al. 2007). Thus, NGF/TrkA signaling serves as a surprising positive regulator which strengthens Ret signaling in a GFL-independent manner (Fig. 2a). Interestingly, TrkA phosphorylation is retained for hours after NGF withdrawal in mature neurons, as compared to the rapid loss of phosphorylated TrkA after NGF deprivation in immature neurons (Tsui-Pierchala and Ginty 1999). Whether this occurs via the same mechanism by which NGF modulates Ret phosphorylation is currently unknown.

The aforementioned studies indicate that, at least in some cell types such as maturing sympathetic neurons, NGF potentiation of Ret signaling can be self-serving wherein Ret reciprocally promotes NGF-mediated trophism, likely through convergence of shared downstream signaling pathways (e.g., MAPK, PI3K). This is likely to be dependent on cell type and on the developmental time period. Within the first 2 weeks after birth, a subpopulation of immature TrkA^+^ DRG nociceptors undergoes differentiation to form two broad populations: peptidergic nociceptors, characterized by their production of calcitonin gene-related peptide (CGRP) and their sustained expression of TrkA, and non-peptidergic nociceptors, characterized by their lack of CGRP expression, their affinity to bind isolectin-B4 (IB4), and their sustained expression of Ret (Molliver et al. 1997). Interestingly, during this diversification process, the emergence of Ret and the GFRα co-receptors is dependent NGF. In DRG nociceptors, Ret promotes the induction of unique gene subsets that are characteristic of non-peptidergic nociceptors, as well as autoregulation of its GFRα co-receptors (Luo et al. 2007). Importantly, Ret was also found to promote postnatal extinction of TrkA expression in these neurons, thereby promoting further diversification of these two populations to yield two molecularly and functionally distinct classes of nociceptors (Luo et al. 2007). Taken together, during postnatal development, there are at least two very different mechanisms of reciprocal signaling between TrkA and Ret. In mature sympathetic neurons, NGF drives Ret autophosphorylation, and not GFLs, which in turn augments the trophic effects of NGF, in contrast to postnatal DRG neurons in which NGF drives Ret expression and GFLs promote Ret activation, which in turn downregulates TrkA expression and upregulates genes necessary for the diversification of non-peptidergic nociceptive neurons (Fig. 2a, b).

### Neurotrophic factor receptor crosstalk: p75 as a rheostat for GFL-Ret signaling

p75, also known as the p75 neurotrophin receptor (p75^NTR^), is an enigmatic receptor whose functions depend heavily upon the cellular context of activation. Although originally identified as a receptor for NGF, p75 is now appreciated to bind all four neurotrophins with high affinity, as well as the unprocessed neurotrophins (pro-neurotrophins) with higher affinity (Ibanez and Simi 2012). In addition, p75 can augment survival signaling of all three Trk receptors, although the mechanism underlying this phenomenon remains incompletely understood. Interestingly, building on the above mechanism wherein TrkA promotes the expression of Ret, which subsequently promotes extinction of TrkA, p75 was recently discovered to be critical in this process. Conditional deletion of p75 in sensory neurons beginning at E12.5 using *Islet1*-Cre led to a significant loss of Ret-dependent non-peptidergic nociceptors by adulthood, without affecting survival of any Trk-dependent DRG neuron populations (Chen et al. 2017). These losses were particularly drastic in non-peptidergic subpopulations expressing low levels of Ret (MrgA^+^ and MrgB^+^) where greater than 50% of neurons were lost, as opposed to the Ret^high^ MrgD^+^ population which lost only ~ 25% of neurons (Chen et al. 2017). Ret^+^ mechanoreceptive neurons that emerge embryonically were not affected, similar to the other mechanoreceptive populations (Chen et al. 2017). p75 is required for GDNF- and NRTN-mediated survival in Ret^+^ non-peptidergic nociceptors in vitro. Consistent with this, p75 is required GDNF-mediated Ret activation and for the cell surface localization of Ret in cultured DRG nociceptors (Chen et al. 2017). Thus, in the reciprocal crosstalk between TrkA and Ret, p75 appears to act as a rheostat to fine-tune GFL-Ret signaling (Fig. 2b). In this model, p75 potentiates Ret activation and downstream signaling through enhanced cell surface routing, which is of particular consequence to neurons that have low basal levels of Ret expression.

### Neurotrophic factor receptor crosstalk: signal integration and coincidence detection in neuronal survival and death

In addition to the trophic functions of p75 in potentiating Ret and Trk signaling as a co-receptor, a plethora of literature also supports the notion that p75 is also critically involved in programmed cell death (PCD) (Ibanez and Simi 2012). In mice lacking p75, the number of sympathetic SCG neurons is substantially increased, and the rate of apoptosis following NGF deprivation is diminished (Bamji et al. 1998). One model that has emerged to explain the temporal kinetics of PCD in the SCG is the “competition factor hypothesis,” in which neurons receiving adequate trophic support (NGF) are protected from cell death and themselves upregulate and release pro-apoptotic p75 ligands which induce apoptosis in nearby neurons receiving inadequate trophic support (Deppmann et al. 2008). In the SCG, BDNF and proBDNF have been demonstrated to trigger apoptosis, owing to the lack of TrkB expression (Bamji et al. 1998; Deppmann et al. 2008; Teng et al. 2005). Interestingly, Ret is expressed in embryonic SCG neurons during PCD and, using a pulse-chase genetic strategy to label Ret^+^ neurons, it was discovered that these Ret^+^ neurons are rapidly eliminated (Donnelly et al. 2018). p75 and Ret physically associate, both in vitro and in vivo, and this interaction is enhanced by pro-apoptotic p75 ligands (Donnelly et al. 2018). Conditional deletion of Ret from primary sympathetic neurons in vitro enhances NGF-mediated survival and inhibits p75/BDNF-mediated apoptosis. Critically, tamoxifen-triggered conditional deletion of Ret or p75 (selectively in Ret^+^ neurons) during PCD significantly reduced apoptosis and increased SCG neuron numbers, indicating that Ret and p75 collaborate in vivo to induce the apoptosis of “unsuccessful” sympathetic neurons (Donnelly et al. 2018). Mechanistically, Ret potentiates apoptosis through two distinct but interrelated mechanisms. First, Ret inhibits TrkA activation and downstream signaling via enhancing TrkA ubiquitination and subsequent degradation (Donnelly et al. 2018). Second, Ret enhances intramembranous cleavage of p75, potentiating activation of downstream pro-apoptotic effectors (Fig. 2c) (Donnelly et al. 2018). The functions of Ret in sympathetic neurons during PCD represents another example of Ret-mediated antagonism of TrkA signaling, as in DRG neuron diversification, underscoring the utility of this mechanism in both neuron differentiation and apoptosis (Fig. 2). In addition, the observation that a common p75-Ret signaling complex can promote either survival or apoptosis also aptly emphasizes the importance of cellular context (e.g., cell type, developmental stage, and trophic status). During PCD in the SCG, Ret functions essentially to “push neurons over the edge” that are not getting sufficient NGF support. As NGF signaling declines in these neurons, Ret is upregulated, thereby making p75 more effective at triggering apoptosis while at the same time inhibiting any remaining TrkA signaling. The apoptotic function of Ret in developing sympathetic neurons raises the question of whether Ret acts as a dependence receptor, in which the binding of ligand (GFLs) induces kinase activity and downstream signaling cascades leading to survival, but in the absence of ligand Ret actively signals apoptosis (Bordeaux et al. 2000). While this is an alluring mechanism, in this context, Ret appears to act in concert with p75 to enhance p75-dependent apoptotic signaling. On a related note, it has recently been reported that the tyrosine kinase activity of TrkA is critical for apoptosis triggered by NGF withdrawal from sympathetic neurons (Feinberg et al. 2017), and whether Ret kinase activity is required its apoptotic function is an important unanswered question.

## Summary

Trafficking GFL receptor components into, or out of, the plasma membrane has a potent influence on the function of these neurotrophic factors. Along with the regulation of Ret expression and degradation, these modulatory mechanisms are pervasive and critical for precise regulation of Ret signal transduction. Thinking of the GFL receptor complex not as a single entity, but as an essential part of a network of receptor crosstalk, may enable a more accurate view of how different cell types respond to combinations of growth factors during development and adulthood. These complex signaling interactions should also be considered when evaluating the potential physiologic and pathophysiologic roles of Ret, Trks, and p75 in cancers and neurodegenerative disorders.
